# Later School Start Time: The Impact of Sleep on Academic Performance and Health in the Adolescent Population

**DOI:** 10.3390/ijerph17072574

**Published:** 2020-04-09

**Authors:** Valentina Alfonsi, Serena Scarpelli, Aurora D’Atri, Giacomo Stella, Luigi De Gennaro

**Affiliations:** 1 Department of Psychology, University of Rome Sapienza, 00185 Rome, Italy; valentina.alfonsi@uniroma1.it (V.A.); aurora.datri@uniroma1.it (A.D.); 2 IRCCS Fondazione Santa Lucia, 00179 Rome, Italy; serena.scarpelli@uniroma1.it; 3 Department of Education and Human Sciences, University of Modena and Reggio Emilia, 42121 Reggio Emilia, Italy; giacomo.stella@sosdislessia.com

**Keywords:** sleep, learning, school start time, academic performance, school health, sleep loss, adolescence

## Abstract

The crucial role of sleep in physical and mental health is well known, especially during the developmental period. In recent years, there has been a growing interest in examining the relationship between sleep patterns and school performance in adolescents. At this stage of life, several environmental and biological factors may affect both circadian and homeostatic regulation of sleep. A large part of this population does not experience adequate sleep, leading to chronic sleep restriction and/or disrupted sleep–wake cycles. Studies investigating the effects of different sleep–wake schedules on academic achievement showed that impaired sleep quality and quantity are associated with decreased learning ability and compromised daytime functioning. This review focuses on the most recent studies that evaluated the effects of modified school start time on sleep patterns and related outcomes. Moreover, based on the available empirical evidence, we intend to propose a direction for future studies targeted to implement prevention or treatment programs by modifying sleep timing.

## 1. Introduction

The pivotal role of sleep in learning and health has been deeply recognized, especially in the context of adolescence [1], one of the most sensitive phases of human development. 

The World Health Organization (WHO) defines adolescents and young adults as individuals aged between 10 and 24 years. Adolescence is a critical period of biological and social changes characterized by dramatic transformations in cognitive, behavioral, and emotional functioning. As expected, there are also significant changes in the sleep–wake pattern.

Over the past century, several studies have consistently described a tendency towards delayed sleep phase, expressed by delayed bedtime and rise time [2,3]. A specific combination of intrinsic (physiological) and extrinsic (environmental) contributing factors leads to a condition of chronic sleep loss in children and adolescents [4]. As a direct consequence of such sleep restriction, a multitude of adverse outcomes affects the quality of life of this population, especially compromising health and daytime functioning. 

Since inadequate sleep among adolescents and young adults represents an alarming and endemic health issue affecting this population, an increasing number of school systems worldwide have implemented later school start time programs to counteract the negative impact of this phenomenon. 

The investigation of school start time effects on adolescent sleep, learning, and well-being has received much scientific effort during the past twenty years. Although most studies have consistently indicated the overall effectiveness of these programs, research on this issue is plagued by some methodological biases. Furthermore, the application of these protocols is challenging for several reasons, mainly linked to social and monetary costs of such policy change. 

The purpose of this paper is to provide an up-to-date overview of current findings on the topic and a critical evaluation of available literature. More specifically, we intend to give some helpful insights and suggestions for planning future studies and for implementing an acceptable school schedule change.

Studies on delayed school times have largely examined the effects on students of elementary, middle, and high schools. However, since the main biological changes in homeostatic and circadian processes covary during puberty, the studies described in the current paper concern the adolescent population.

In this review, we outline the latest studies on the consequences of delaying school start times, focusing on primary effects on sleep (wake-up time, bedtime, total sleep time, daytime sleepiness) and secondary effects on daytime functioning (school performance, vigilance levels, well-being, and risk-taking behavior). To introduce this primary issue, we briefly depict the most recent findings on current sleep condition in adolescents within a consolidated theoretical framework. Then, we also explore the relationship between adolescent sleep loss and principal domains of human functioning (mental and physical health, cognitive and behavioral performance). Finally, we critically discuss studies investigating the effect of later school start time programs, highlighting strengths and weaknesses of these studies and the potential benefit–cost ratio of the application of this public intervention. 

## 2. Methodological Note

The literature search was conducted using PubMed queries and reference lists of the selected studies. Key search terms included:

“Adolescence” AND “Sleep” AND “Circadian process OR Homeostatic process” (432 articles);
“Adolescence” AND “Sleep deprivation” (80 articles);
“Adolescence” AND “Sleep” OR “Sleepiness” AND “School timing” (170 articles);
“Adolescence” AND “Health” OR “Academic performance” AND “School Timing” (236 articles).

Search terms had to be included in the abstract, the title, or the keywords. We did not limit results to the year of publication. We considered the available studies up to December 2019.

We grouped the identified citations in the following categories:

1. Sleep pattern, sleep homeostasis, and circadian rhythms in teenage students;
2. Adolescent sleep loss and negative consequences on (a) mental and physical health, (b) learning abilities and academic performance, (c) risk-taking behavior;
3. Experimental evidence on delayed school start time.

We focused on the effects of adolescent sleep deprivation and the impact of delaying school start time, excluding non-English articles and including the peer-reviewed published papers. All the articles resulting from using these selection criteria and related to our focus were included. Following this method, 127 publications were estimated to be of interest for further examination and were included in this review.

## 3. Sleep in Adolescents: How They Sleep and How They Should Sleep 

The main alteration in sleep patterns throughout this period refers to a delay in the timing of sleep phase; adolescents tend to fall asleep and wake up later than children and adults [2,3]. This phenomenon is traditionally described within the theoretical framework of the two-process model proposed by Borbély in 1982 [5], based on the reciprocal interaction between circadian and homeostatic processes of sleep regulation. 

Circadian rhythms are driven by an internal clock, situated in the suprachiasmatic nucleus (SCN) of the anterior hypothalamus. In addition to this endogenous pacemaker, several external conditions entrain the circadian system. The major synchronizer is represented by environmental light, which maintains the circadian period close to 24 h. The light signal received through the retina is transmitted to the SCN and finally to the pineal gland, which produces melatonin, the hormone associated with sleep onset and frequently used as a phase marker.

The available literature on melatonin release in children and adolescents is scarce and inconsistent. Some studies examined nocturnal melatonin patterns and found a global reduction in the basal levels of melatonin during the pubertal stage [6,7], in contrast to previous studies [8,9]. Recent data have also indicated that melatonin seems to exhibit greater sensitivity to evening light [10,11] and less sensitivity to morning light [12] in young people than adults. Overall, these results emphasize the potential relevance of phase-delaying effects of light stimulus in this population. Coherently with these findings, the circadian chronotype shifts from morningness (up to 10 years of age) to eveningness in adolescence, and then return towards morning chronotype in advanced age (after 50 years of age) [13,14,15]. 

The circadian chronotype is also affected by other factors, such as gender. In particular, females shift toward eveningness earlier than males (17 vs. 21 years of age) [15], consistently with their early onset of pubertal development. 

Recent studies have focused on the other aspect of sleep timing co-occurring with circadian sleep patterns—the homeostatic sleep drive. According to the two-process model of sleep, the pressure to sleep increases as an exponential function during waking period, and, on the contrary, it progressively dissipates during sleep. Spectral power in the low electroencephalography (EEG) frequency band (~0.75–4.5 Hz) represents a sensitive marker of the homeostatic process, showing an increase dependent on previous waking period and a gradual decay throughout sleep episodes. 

Preliminary findings on the homeostatic sleep dynamics revealed the absence of any significant variations in sleep pressure decrease between pre- and post-pubertal adolescents, as reflected by the overlapping decline of slow-wave activity (SWA) across sleep cycles [16,17]. These results are corroborated by recent studies using longitudinal measurements [18] or a different methodological approach [19]. On the other hand, the increase of homeostatic sleep pressure—reflected by the build-up of SWA—exhibits a slower rise rate in mature adolescents than in prepubertal children [17]. To sum up, sleep needs and recovery processes are unmodified during adolescence. In contrast, lower sleep pressure and higher tolerance to stay awake at the end of the day characterize this population. 

This evidence parallels changes resulting from the circadian process alteration, emphasizing the relevant contribution of both circadian and homeostatic regulation also in the expression of sleep/wake cycle changes typical of adolescence. 

Data collected in several studies described the presence of a sleep phase delay during adolescence in different countries, independently of substantial cultural variations in their habits over the world [20]. Such cross-cultural homogeneity suggests a biological basis in the predisposition to go to bed and get up later. This notion is also supported by studies on neurodevelopmental trajectories in the adolescent brain. Besides the phase shift in circadian rhythms and the dysregulation in the homeostatic process, sleep physiology undergoes many maturational changes during adolescence [21,22]. The most explicit expression of sleep EEG modification is the reduction of EEG amplitude and power (up to 40%) across EEG frequencies [23,24]. Specifically, SWA shows an increase from birth to the beginning of adolescence and a subsequent reduction throughout puberty (inverted U-shaped curve) [16,25,26,27]. At the same time, the EEG coherence during sleep shows a linear increase across the subsequent sleep cycles [28]. Such functional modifications in the oscillatory physiology are probably linked to structural changes in the adolescent brain. The reduction of grey matter volume, associated with local synaptic pruning, is observed in the teen years, and it could be related to the decline of EEG activity, as demonstrated by neuroimaging studies [29]. In the same way, strengthening of the EEG connectivity may be driven by the increase of white matter volume and myelination process, which takes place during puberty [22]. These anatomical modifications could also be associated with the rise of peak spectral frequency of sleep spindles during adolescence [30], as a marker of cortical myelination [31]. 

Adolescents struggle to fall asleep early because of a combination of bioregulatory processes and external factors related to modern lifestyle. Carskadon and colleagues proposed that the intrinsic tendency of adolescents to go to sleep and wake up at late hours could “open the gate” for evening or night activities [4]. Several extrinsic factors allow them to stay active and contribute to their sleep loss, including social life and engagements late in the evening or night, increase in homework, afterschool activities, and, above all, the use of electronic media (television, computer, mobile phone) during the night [32,33,34]. Light plays a primary role in synchronization of the human circadian system. The long-term night-time exposure to bright light in adolescence leads to inhibition of melatonin secretion and to a consequent delay in falling asleep [35]. In summary, some dysfunctional behaviors could be exacerbated by a biological predisposition and, in turn, produce a further deterioration in sleep quality. Carskadon and colleagues [4] described this process with a model called “The Perfect Storm”, illustrating the detrimental and the cumulative effects of biological, psychological, and social factors on sleep. 

The adolescent’s nightly sleep need is around 9–9.35 h for optimal health and functioning [36,37]. Based on empirical evidence, many medical organizations, including the American Academy of Sleep Medicine, American Academy of Pediatrics, Sleep Research Society, and American Association of Sleep Technologists, recommend that adolescents (13–18 years of age) should regularly sleep 8–10 h per night to promote an adequate health and performance [38]. However, delayed biological bedtime and early awakening due to school attendance inevitably result in a condition of chronic sleep debt in this population. Despite the recommendations, most adolescent students (about three quarters) report sleeping less than 8 h per night [39], and this amount increases as a function of school grade level. 

Epidemiologic studies conducted in Europe, Asia, and the United States in the teenage years suggest inadequate sleep in many adolescents (from 6% to 37%) [40], which is reflected in difficulties at the beginning of sleep, through the night, and towards the end of night sleep [41,42,43]. Adolescents also have increased levels of daytime sleepiness [44,45] and frequent diagnosis of insomnia [46,47]. Moreover, morning rising time during school days leads to a large discrepancy between weekday and weekend sleep patterns as a direct consequence of rebound sleep on non-school days due to accumulated sleep debt during the week [48,49].

## 4. The Effects of Sleep Deprivation on Health, Performance, and Behavior

Over the last two decades, our knowledge about the critical role of sleep in well-being and brain function of the adolescent population has notably increased. 

Insufficient sleep in the teenage years has been tied with a wide range of adverse outcomes affecting their lifestyle. Three main areas of daytime functioning are affected by chronic sleep restriction: mental and physical health, cognitive and academic performance, and risk-taking behaviors. 

There is a solid body of literature pointing to a strong link between sleep quality and physical health. The main physical health consequences of adolescent sleep loss refer to metabolic dysregulation and cardiovascular morbidity. For example, an increase in body weight [50], a higher risk of obesity [51,52,53], and a reduced physical activity [54] in association with low sleep were observed in the teen population. Furthermore, sleep loss in adolescents is likely to lead to increases in blood pressure [55,56] and high cardiometabolic risk [57]. Poor sleep in adolescents is also positively associated with other somatic outcomes, such as headache [58], persistent fatigue [59], and lower back, neck, and abdominal pain [60].

Given the well-established relation between sleep and many psychiatric disorders such as depression or anxiety [61], the side effects of adolescent sleep debt on mental health are not surprising. More specifically, empirical studies showed high odds of depressive symptoms among adolescents with insufficient sleep duration [62,63,64,65]. Another serious source of concern is represented by the elevated rate of suicidal ideation [66,67,68] or suicidal attempts [69] in sleep-deprived adolescents. A possible explanation for the key role of sleep in the onset of mental disorders could be the physiological alteration of mood and emotional regulation as a result of acute or chronic sleep deprivation [70,71]. 

Experimental studies applying sleep restriction protocols demonstrated the worsening of several neurocognitive functions, such as memory, attention, and executive functions, as a consequence of sleep loss [72,73,74]. In particular, the major impediment referred to circumstances requiring multi-tasking skills [75] frequently faced by young people. 

Naturally, these harmful effects on cognitive functioning impair their academic performance. Several prospective and cross-sectional studies supported the notion of a strong correlation between scarce sleep quality and low school achievement [76,77,78,79,80]. However, the modulatory effect of individual and environmental factors could explain some negative findings [76,81,82]. As previously described, adolescents have a natural circadian preference for evening chronotype. A recent study on a large sample compared the two extreme chronotypes and found lower school grades in “evening type” compared to “morning type” adolescents [83].

Several studies described a positive relationship between inadequate sleep and engagement in risk-taking behavior in adolescents, especially with regards to substance abuse [84]. Insufficient sleep was linked to greater tobacco smoking and marijuana use [85], alcohol consumption [86], and abuse of other illegal drugs [68]. Sleep loss was further associated with unhealthy behavioral strategies [87], bullying [88], physical violence [63], and unsafe sexual activity [89]. 

Excessive sleepiness due to sleep restriction represents the main reason for motor vehicle accidents in the adolescent population, especially in the context of late-night or early-morning driving [90]. A growing number of studies report increased car crashes in sleep-deprived adolescents [91,92,93,94]. Since motor vehicle accidents represent the principal cause of mortality among youths in the United States [95], the high crash rate in this age group constitutes a matter of great concern. 

Notably, the nature of the relation between health, cognition, behavior, and sleep is often bidirectional [96]. Therefore, intervening on sleep patterns could engender a cascade of positive outcomes on other areas of functioning. The exact role attributable to sleep restriction is difficult to establish. However, a wide variety of studies show the crucial relevance of poor sleep, especially in a transitional stage such as adolescence. Indeed, problematic conditions arising in this period often increase the likelihood of developing and then chronicizing future disorders in adulthood [97].

## 5. Benefits and Challenges of Delayed School Start Times

Insufficient sleep in adolescents represents a major public health issue [98]. As extensively described above, chronic sleep restriction results in many adverse consequences on both nocturnal sleep and daytime functioning. Sleep–wake schedules set by school do not fit with biological circadian and homeostatic processes regulating adolescent sleep patterns. Consequently, several aspects of quality of life and education of teenage students are compromised [99]. The American Academy of Pediatrics has recommended that middle and high schools should begin no earlier than 8:30 a.m. to adequately satisfy the sleep needs of students. 

A large number of school systems all over the world have applied later start time programs as a policy change to reduce the imbalance between early waking time and adolescent sleep phase delay in order to address adverse outcomes intervening on the main external factor of sleep curtailment in this population. Indeed, although delayed sleep onset is primarily related to intrinsic factors, it is possible to intervene on school schedule directly. 

The pioneering empirical study in this field was conducted by Carskadon and colleagues [3]. They estimated changes in sleep patterns, sleepiness, and circadian phase across the transition from 08:25 a.m. start time (9th school grade) to 07:20 a.m. start time (10th school grade). They showed for the first time a reduction of sleep duration (about 20 min) and an increase of daytime sleepiness associated with earlier school start times. 

Preliminary findings on the beneficial effects of delaying school start time (mostly focused on sleep-related outcomes) [100,101,102] were followed by subsequent studies described in this review, illustrating the secondary effects on performance and behavior. To date, the body of work on later school times is extensive and constantly growing, confirming the early work by Carskadon et al. [3]. 

The principal results of the key studies are summarized in Table 1. Since delaying school start times is primarily intended to address the problem of insufficient sleep among adolescents, most studies focused on the association between school start times and sleep variables. 

Recent systematic [103,104,105] and meta-analytic [106] reviews provided a qualitative and quantitative synthesis of the strength of delayed school programs in improving several sleep-related outcome variables. The outcomes, mostly estimated by sleep habits survey, illustrated the effectiveness of these programs on rising time, bedtime, or time spent in sleeping. As expected, a significant delay in wake time of students with later start times was observed in an overwhelming majority of studies [74,107,108,109,110,111,112,113]. Specifically, there is substantial empirical evidence that later rise time varies from a minimum of 21 to a maximum of 61 min [105]. 

On the other hand, studies evaluating the effects on bedtime showed mixed evidence. Sometimes later [108,114,115] or earlier bedtime [109,112] was observed post-intervention. Otherwise, most results indicated the absence of significant variations [74,107,110,111,116]. Therefore, there is no clear association between school start times and bedtime, contrary to the simplistic hypothesis that students tend to delay falling asleep as a result of a clock time delay. 

Taken together, most cross-sectional and longitudinal studies reported an increase in total minutes of sleep following later school start times [103]. The grade level in school (elementary school, middle school, high school) was not a predictor of any variations in the magnitude of increased sleep [106], suggesting the maintenance of effect regardless of the specific school grade. 

As previously described, it seems evident the phenomenon of weekend oversleeping due to progressive deprivation accumulated during weeknights in teenagers. The nature of changes following the delayed school time program suggests that sleep debt during weeknights is reduced in the intervention group [109,110,116,117,118]. 

Another main problem related to sleepiness is the tendency to fall asleep and napping during school hours, with an obvious negative impact on academic success. Delaying school start times can be a possible method to minimize the difficulty of staying awake during lessons by reducing the levels of daytime sleepiness. The effects on sleepiness have been found in several studies, showing differences between estimated daytime sleepiness in the intervention and the control group. Not surprisingly, students with later start times (and longer sleep durations) appeared to decrease their level of daytime sleepiness in all studies conducted, except for two [116,119]. 

Delaying school times is also tied to secondary outcomes for teenage students. These programs have been implemented with the express purpose of reducing the negative impact of early school times on student well-being and performance by improving the sleep–wake schedule. Observing the secondary effects of sleep extension on health, behavior, and cognition may aid in understanding the extent of the benefits of later school times. 

In their longitudinal studies, Wahlstrom and colleagues [107,120] found better academic performance in the later start times group, in agreement with other works [121,122,123]. Otherwise, two studies did not reveal significant effects of intervention in self-reported academic outcomes [109,110] or a specific effect dependent on school-grade [108]. 

Attention and vigilance are closely related to learning ability and cognitive function and, therefore, to success in school. Significant enhancement of attention level during class [74] along with faster reaction time [116] were observed in the later start times sample. 

Another result of great interest relates to the impact on school attendance and tardiness. Studies examining the effects of starting times on absences from school did not indicate significant variations related to starting times [108,113,124]. Conversely, samples of students with delayed start times showed a considerable reduction of marks for lateness [109,110,124]. 

Given the strong relationship between sleep and physical and mental well-being, later start times studies also evaluated changes in health-related behavior. Most of the cases showed an increase of positive mood and affect [109,110,113] and a decrease in school absenteeism due to illness [123]. One study measured mental health indicators and reported fewer depressive symptoms in students with later start times than in the control group, although these differences were not significant [107]. Likewise, reduced healthcare utilization was reported following the intervention [109]. 

The school time delay also led to variations of other aspects related to physical health, such as body mass index changes [125] or caffeine consumption [110]. Specifically, weight loss and reduced caffeine use were associated with the intervention. 

Furthermore, empirical evidence points to a positive association between later school start times and reduction of vehicular accident rates (by 16.5%) [120,126,127,128], potentially by improving vigilance. 

## 6. Limitations and Future Directions

This review outlines the principal evidence concerning the consequences of chronic sleep deprivation in adolescence, focusing on studies examining the effects of later school time.

Investigations on school start times are of two types: cross-sectional and longitudinal studies. The first category refers to studies observing the effects by comparing the outcomes observed in schools (or classes) having different start times. The second one includes studies comparing the data collected before and after the application of later school programs on the same student group. Clearly, longitudinal studies have the advantage of showing the extent of change through within-subject comparisons. However, this study design does not consider the spontaneous variations simply due to the passage of time.

The available literature does not provide a homogeneous methodological framework. Nonetheless, the discussed results emphasize an overall positive effect following this policy change. Marx and colleagues [130] proposed two distinct explanations underlying the widespread benefits of school start time interventions; one refers to the net increase of amount or quality of sleep, and the other is related to the existence of an optimal time of day for vigilance and learning potential.

Most studies showed the positive effects of delaying school start time initially on sleep patterns and secondly on daytime functioning. Therefore, just as the endemic sleep loss in adolescents triggers negative consequences through a “vicious cycle” [35], delaying rise times could, in turn, generate positive outcomes on sleep and lifestyle through a “virtuous cycle”.

As mentioned above, the existing literature shows several weaknesses from a methodological standpoint. It is noteworthy that nearly all studies used self-report instruments (questionnaire, survey, diaries) in evaluating the impact of changing school start times, except for a few studies using actigraphy [3,74,117,131] or polysomnography [3] to objectively measure sleep. Recently, in the study by Dunster and colleagues [117], the effect of an across-the-district change in school start times on sleep was objectively quantified for the first time. Notably, delaying start times (one hour) produced an increase in sleep duration of nearly half an hour, as measured by wrist actigraphy.

Almost all studies to date varied in their sample size and composition, ages of participants, and experimental design. Furthermore, most studies lack the comparison with an adequate control group, preventing attribution of the observed changes to school start time factor. As an example, the recent study by Chan and colleagues [113] includes a control group with no adjustment in school start time. However, the control school does not represent a valid comparison group because the two schools had no comparable school schedules at the beginning of the intervention with obvious limitations in interpreting their results.

Another critical issue about this literature refers to the exact quantification of how much time (minutes) school start should be delayed for ensuring the desired effect. In a recent meta-analysis [108], the length of delay was associated with the extension of sleep duration in a positive direction. In other words, the longer the delay is, the greater is the lengthening of total sleep time (TST). The specific conditions were not standardized across studies, ranging from a minimum of 20 to a maximum of 60 min of delay. However, a 60 min change between early and later start times seems to be a critical threshold in determining the effects [104], as shown by a net gain of TST when the delay was greater than 60 min [108,110,124,129]. Generally, the increase in minutes of sleep ranges from 25 min to 77 min per night [105]. Most evidence is based on intervention studies with shifted start times no later than 09:00. Recently, the study by Kelley and colleagues [123] went beyond the clinical recommendation that middle and high school should start later than 08:30 and examined possible additional benefits of moving starting times from 08:50 to 10:00 (1:10 h delay). They found a considerable reduction of absences due to illness (by over 50% compared to national rates) and a significant improvement in academic performance. However, the lack of sleep measures in this study makes it difficult to interpret the actual impact of intervention, since we cannot directly link positive outcomes to changes in sleep duration or quality.

Furthermore, numerous studies were conducted without any follow-up protocols. A recent study conducted in a Singapore school [131] investigated the effect of changing start times at 1 month and 9 months after the intervention start. The authors showed sustained gains on sleep duration, daytime alertness, and mental well-being. On the other hand, the beneficial outcomes were not maintained over time in other studies [124]. Different methodological approaches in each study could explain these contradictory results. Another potential reason could be linked to the possibility that delayed wake-up time allowed adolescents to progressively shift their bedtime as well, as observed in a recent follow-up study by Rhie and colleagues [118]. Their observational study demonstrated a transient increase in sleep duration with a reduction of the initial effect over time parallel to a delayed circadian cycle. Determining the long-term effectiveness of intervention represents a crucial factor for a stable implementation of this policy shift. Therefore, future follow-up studies are highly desirable because of both practical reasons and inconsistent results.

Different school start times are associated with many variables related to sleep, health, and academic performance. However, it is important to note that the correlational nature of the relationship between observed variables does not necessarily imply causality; therefore, results must be interpreted with extreme caution. Studies exploring the consequences of delayed school start time in the academic context revealed no clear association between start times and school performance. An intrinsic limitation of this kind of study is the absence of standardized test scores and the lack of randomized controlled trials [103].

An additional aspect closely related to both academic performance and sleepiness is the effect on sustained attention or vigilance, which has never been directly investigated. On this basis, some insights for incoming studies seem opportune. For example, it might be useful to consider using validated and feasible tests measuring reduced behavioral alertness due to sleep loss, such as the Psychomotor Vigilance Task [132,133].

Future studies should also investigate the effects on the well-known phenomenon of sleep inertia, occurring during the transition from sleep to wake [134,135]. Sleep inertia affects several cognitive and sensory-motor abilities, and delaying school start time could interfere with the physiological performance impairment due to this phenomenon. 

Along with the methodological limitations described above, this kind of study presents an array of complications related to the implementation costs of changing school start times. The benefits of delaying school start times are well-documented, but several economic and logistic problems remain unresolved. 

The main financial concern refers to the changes in school bus schedules. The cost assumption is strictly dependent on the specific characteristics of the district (e.g., rural or urban), and it is mainly related to both potential overlap with elementary or middle school bus systems and eventual increase of traffic. However, one study analyzing the benefit–cost ratio suggested that shifting school start times represents a cost-effective strategy in the long term, resulting in substantial economic benefits relative to costs [95]. 

The need for restructuring daily activities represents another great concern. Adequate planning is necessary to ensure the proper execution of extracurricular and sports activities, fundamental for an adolescent’s well-being. In this respect, it is worth pointing out that the study by Chan and colleagues [113] found positive effects following the shortest delay intervention applied (15 min), which could be considered a plausible compromise.

Additional research is certainly needed. However, given the relevant societal burden represented by chronic sleep loss in the adolescent population, the involvement of stakeholders (students, parents, teachers, school bus drivers) is necessary to improve current school policies and develop the future ones. Sleep hygiene education programs represent a possible countermeasure to address this issue, especially in East Asian societies, where academic success is a priority over sleep quality. These programs generally consist of a set of behaviors (e.g., regular sleep schedule and routine, moderate use of caffeine, reduction of naps, avoidance of stimulating activities at night) to establish a good sleep practice. Although these programs are effective and useful in raising subjective awareness, their exclusive application has scarce and low effects on adolescent behavior [136]. As a possible solution, the combined application of sleep hygiene and delayed start times programs [137,138] could represent an effective solution in addressing adolescents’ sleep problems by increasing individual motivation and providing a concrete countermeasure to short sleep duration.

In summary, we propose the following possible directions for further research in this field:

- The outcomes of delayed school start times should be investigated using both subjective and objective measures;
- Future follow-up studies are needed to establish the long-term effectiveness of the intervention;
- The effect of the intervention on sustained attention or vigilance should be addressed;
- Future studies should examine the impact of changing school start times on phenomena related to sleepiness and vigilance, such as sleep inertia;
- The combined application of sleep hygiene and delayed start times programs could represent an effective solution.

## 7. Conclusions

Insufficient sleep among school-aged adolescents has become an alarming health issue. Numerous studies worldwide consistently describe a wide variety of adverse consequences, including health risks, poor cognitive performance, and behavioral accidents. 

In addition to biological and social factors, school timing also contributes to inadequate sleep conditions in this population. Early school start time generates an evident mismatch with the natural phase shift in adolescence, leading to chronic sleep restriction. Besides, many studies showed the benefits of the implementation of delayed school start times programs. 

This review aims to synthesize evidence relating to the well-known phenomenon of sleep loss during adolescence and to provide an up-to-date overview of research on school start times, with the ultimate purpose of giving suggestions for future studies. 

To sum up, we confirm the overall success of delaying school start time and bring to light a twofold problem of this line of research: methodological and practical. 

Unlike previous works [103,104,105,106], systematic and meta-analytic analysis is beyond the scope of this review, and this entailed some basic limitations. Indeed, a rigorous methodological quality review of the included literature was not undertaken, and, consequently, some of the cited studies are less rigorous and have more potential bias than others. 

As a concluding remark, we emphasize the clinical and the educational relevance of research in this field and the need for future studies that take into account the identified challenges.

## Figures and Tables

**Table 1:** Characteristics of the key studies on delayed school start time.

Authors (Year)	Sample Size (School Grade)	Study Design	Measures	Main Results
Wahlstrom (2002) [107]	>12.000 (grades 9–12)	Longitudinal	Sleep	↑ sleep duration ↑ rise time ↓ sleepiness
			Academic performance	↑ school rates ↓ tardies/absences
			Health	↓ depressive mood
Wolfson (2007) [108]	205 (grades 7–8)	Cross-sectional	Sleep	↑ bedtime ↑ rise time ↑ sleep duration ↓ WE oversleep ↓ sleepiness
			Academic performance	↑ school rates ↓ tardies/absences
Owens (2010) [109]	201 (grades 9–12)	Longitudinal	Sleep	↑ sleep duration ↓ bedtime ↑ rise time ↑ sleep satisfaction ↓ sleepiness
			Academic performance	↓ tardies/absences
			Health	↓ depressive mood ↓ HS utilization
Borlase (2013) [112]	667 (grades 9–12)	Longitudinal	Sleep	↑ sleep duration ↓ bedtime ↑ rise time ↓ WE oversleep ↓ sleepiness
Short (2013) [129]	687 (grades 9–12)	Cross-sectional	Sleep	↑ sleep duration
Boerges (2014) [110]	197 (grades 9–12)	Longitudinal	Sleep	↑ sleep duration ↑ rise time ↓ sleepiness
			Academic performance	= school rates
			Health	↓ depressive mood ↓ caffeine use

Abbreviation: WE: weekend; HS: healthcare service; Legend: ↑: “increase” (sleep duration, sleep satisfaction, WE oversleep, sleepiness, school rates, tardies/absences, depressive mood, HS utilization, caffeine use) or “delay” (bedtime, rise time); ↓: “decrease” (sleep duration, sleep satisfaction, WE oversleep, sleepiness, school rates, tardies/absences, depressive mood, HS utilization, caffeine use) or “advance” (bedtime, rise time).
